# High Sulfate Attack Resistance of Reinforced Concrete Flumes Containing Liquid Crystal Display (LCD) Waste Glass Powder

**DOI:** 10.3390/ma12122031

**Published:** 2019-06-25

**Authors:** Seong-Kyum Kim, Won-Kee Hong

**Affiliations:** 1 Department of Civil Engineering, Kumoh National Institute of Technology, Gumi-si, Gyeongsangbuk-do 39177, Korea; skim@kumoh.ac.kr; 2 Department of Architectural Engineering, Kyung Hee University, Yongin-si, Gyeonggi-do 17104, Korea

**Keywords:** liquid crystal display (LCD), waste glass powder (LWGP), sulfate attack resistance, reinforced concrete flumes, flexural load, Korean industrial standards (KS)

## Abstract

To prevent chemical erosion of concrete and improve chemical resistance, reinforced concrete flumes were manufactured, conforming to the Korean Industrial Standards (KS). Two different sizes of liquid crystal display (LCD) waste glass powder (LWGP) particles were used (i.e., 5 and 12 µm) with two substitution types with cement in concrete (i.e., 10% and 20%). Changes in compressive strength, pore structure, weight, volume, and strength of the concrete flumes after immersion in two sulfate solutions (i.e., Na_2_SO_4_ and MgSO_4_) for 84 and 182 days were measured for sulfate attack resistance. The applicability of the LWGP concrete flume with a 0.5 mm crack width was also evaluated based on the bending strength results. The LWGP5, which has a smaller particle size among LWGPs, filled the smaller pores, thereby reducing the porosity and contributing to the compressive strength gain. Higher volume and weight change ratios for all specimens immersed in MgSO_4_ solution were found than those immersed in Na_2_SO_4_ solution under identical conditions. Flexural loads of all the LWGP concrete flumes with 0.05 mm crack widths were greater than 48.5 kN, as required by the KS code; however, these flexural loads were lower than those of ordinary Portland cement. The applicability was also validated via a flexural test complying with KS.

## 1. Introduction

In recent years, technological development of concrete materials has enabled the design of enlarged, high-rise, and multi-functional structures and plays a pivotal role in protecting human lives and properties from various disasters. Concrete structures have been designed and constructed to fulfill the same requirements as those of multi-functional structures. However, the durability of concrete structures in a marine condition and in wastewater treatment facilities in severe environments easily deteriorates from chemical erosion and often causes structural problems [1,2,3]. In particular, reinforced concrete flumes are most commonly used in flow water, sewage, and wastewater plants. The durability of such flumes reduces because of various pollutants contained in water [4,5,6,7,8,9].

Several studies have aimed at incorporating various binders together with cement to prevent chemical erosion of concrete and improve chemical attack resistance. In particular, the incorporation of pozzolanic materials produces a C–S–H phase within the cement matrix, which further densifies the cement matrix. It reduces permeability and improves the durability of concrete by suppressing the pore continuity.

Meanwhile, Korea’s investment in the LCD industry is as high as 27 billion USD per year, with a production of over 480,000 panels per month (the 8th generation, 50 inches) [10]. The composition of LCD waste glass occasionally fluctuates because of the use of different products. Therefore, LCD waste glass recycling is difficult in a field that is sensitive to composition change [11]. As most waste LCD is incinerated or buried, it wastes resources and causes various types of environment pollution on a national scale [12].

LCD contains SiO_2_ as the main component, which is similar to silica fume (SF), fly ash (FA), and blast furnace slag (BS); these components improve the pozzolanic reactivity. The replacement dosage of cement and the high fineness of the LCD waste glass powder (LWGP) are considered appropriate for durability improvement. Although the substitution of cement with LWGP improves durability as well as mechanical and microstructural properties, the deterministic characteristics of LCD glass powder particles include particle size/surface area and the substitution amount [13,14,15,16,17,18].

Herein, concrete containing 10% and 20% of LWGP was mixed with ordinary Portland cement (OPC). Sodium sulfate (Na_2_SO_4_) and magnesium sulfate (MgSO_4_) were used as solution for simulation of the sulfate-bearing environments. Changes in compressive strength, volume, and weight caused by chemical erosion were measured and compared. Based on these results, concrete flumes with an improved chemical resistance were fabricated to conform to the Korean Industrial Standards (KS F 4010) [19]. The applicability of the LWGP concrete flume was then evaluated based on the bending strength results when crack widths of 0.05 mm were first generated. A new concrete channel using LWGP was developed herein, which could increase the resistance of the concrete structures to sulfate attack.

## 2. Materials and Experimental Methods

### 2.1. Material Characterization and Mix Properties

OPC and two different types of LWGP were used as the primary binder in this experimental study. LWGP were prepared by grinding the LCD glass [13]. Table 1 shows the chemical properties of OPC and LWGP. The LWGP, with mean particle sizes of 5 and 12 µm and consisting of SiO_2_ (69%), Al_2_O_3_ (19%), and CaO (10%), was used as the admixture similar to the fly ash used as the concrete binder. In order to minimize the multi-degradation originating from carbonate aggregates (i.e., limestone-based aggregates) on the specimens [20,21], local cleaned river sand passed through 5 mm and crushed stone with a maximum size of 25 mm were used for fine and coarse aggregates, respectively. Its physical properties are presented in Table 2. The LWGP was made out of films with defects, which were collected from the manufacturing process, such as cutting or scribing [22]. They were not collected from the products after use. Borosilicate glass with an average thickness of 0.4 to 1.1 mm, which did not contain heavy metal, was used to make the LWGP. LWGP5 and LWGP12 with different mean particle sizes and a specific gravity of 2.79 and 2.81 replaced every 10% of the weight of OPC up to 20% as shown in Table 3.

### 2.2. Test for Slump of the LWGP Concrete (KS F 2402)

**Figure 1:**
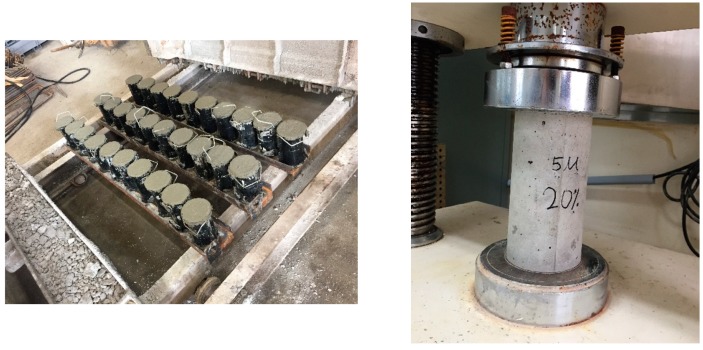
Test to measure the compressive strength.

Concrete slump was checked according to KS F 2402 [25] (Figure 1). The mold shell was used in this test with the following dimension: The top was 100mm in diameter; the base as 200mm in diameter; the height was 300mm; and the thickness was 1.5 mm. The mold was removed immediately from the concrete when filled. Concrete compaction of 25 times was performed for every 1/3 fill, and the height of collapse was measured shortly after the slump cone was removed.

### 2.3. Test for the Compressive Strength of Concrete (KS F 2405)

The concrete specimens shown in Figure 1 were tested to measure the compressive strength according to KS F 2405 [26]. The average strength of three specimens was obtained at 7 and 28 days.

### 2.4. Test for the Porosity and Pore Size Distribution

Mercury intrusion porosimetry (MIP) using Micromeritics (Autopore IV 9500, Micromeritics Instrument Corp., Georgia, USA) was implemented to measure the concrete porosity ratio and distribution of the concrete specimens with a 1.0 cm^3^ volume as a function of the replacement ratios. The water was totally dried out under a 105 °C after the specimens were submerged in an alcoholic solution for a week to halt hydration.

### 2.5. Test for the Sulfate Attack Resistance of Concrete

The cured specimens at 28 days were immersed in a solution of 5% Na_2_SO_4_ and MgSO_4_ to measure the reduction ratios of the volume, weight, and the changes of the compressive strength at 84 and 182 days. The LWGP concrete specimens cured in water for 28 days were immersed for 182 days. The used sodium sulfate and magnesium sulfate solutions were prepared according to the American Society for Testing and Materials (ASTM) C 1012 standards [27]. The solution used in the experiment was replaced by new solution every 30 days of immersion considering factors, such as moisture loss due to evaporation. The temperature of the experiment condition was kept constant at 23 ± 2 °C and the solution was stirred periodically so that the solution concentration in the immersion containers was uniformly distributed.

### 2.6. Manufacturing LWGP Concrete Flumes

**Figure 2:**
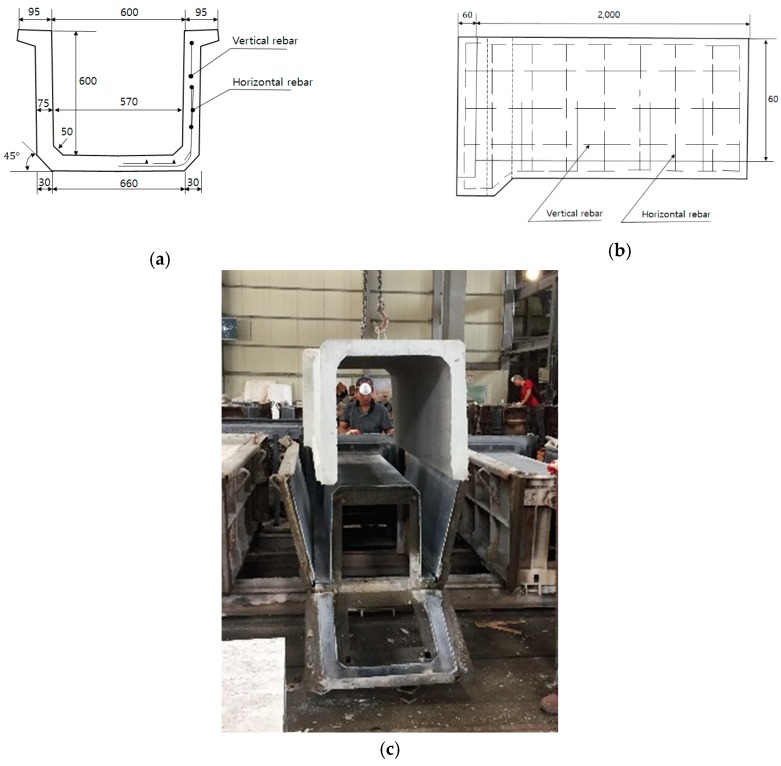
Dimensions and type of LWGP concrete flumes. (**a**) Cross section; (**b**) Longitudinal section; (**c**) Specimens production.

Figure 2 shows that the reinforced concrete flume type Ⅲ identical to those used in the site were manufactured under 600 C (L = 2000 mm) according to KS F 4010 [19]. The 10% and 20% cement weight was replaced by concrete using the LWGP. Manufacturing was done under the curing condition identical to that in the construction site. Figure 2a shows the cross-sectional dimensions of the fabricated specimen and 2b shows the dimensions of the side view. Figure 2c shows the specimens produced at the manufacturing site.

### 2.7. Flexural Loads of the LWGP Concrete Flumes

**Figure 3:**
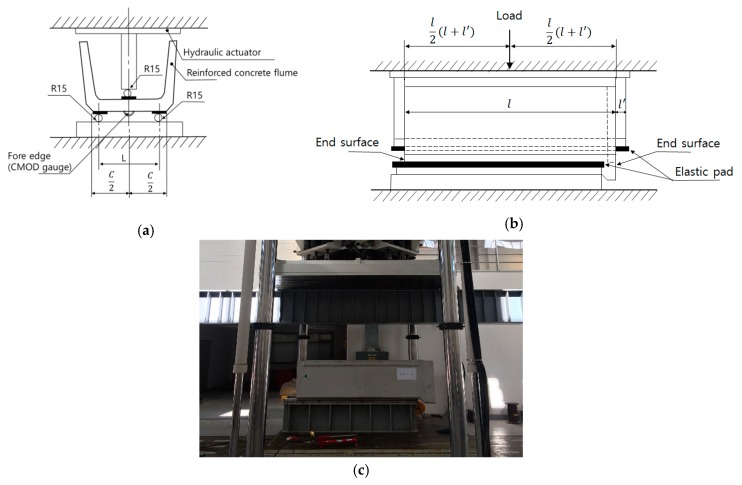
Test method of the LWGP concrete flumes from KS F 4010. (**a**) Cross section; (**b**) Longitudinal section; (**c**) The test setup for the LWGP concrete flume.

Figure 3c shows the test setup for the LWGP concrete flumes. The load was applied at the mid-point of the loading pad, which was installed longitudinally (l = 2000 mm as shown in Figure 3b). The load applied on the loading pad per 1 mm/min was measured. The length of the LWGP concrete flumes was 550 mm (L) according to KS F 4010 [19] under 600 °C. The Crack Mouth Opening Displacement (CMOD) gauge was attached to the end of the LWGP concrete flumes to measure the crack width caused by the loads (Figure 3a).

## 3. Results and Discussion

### 3.1. Slump

**Figure 4:**
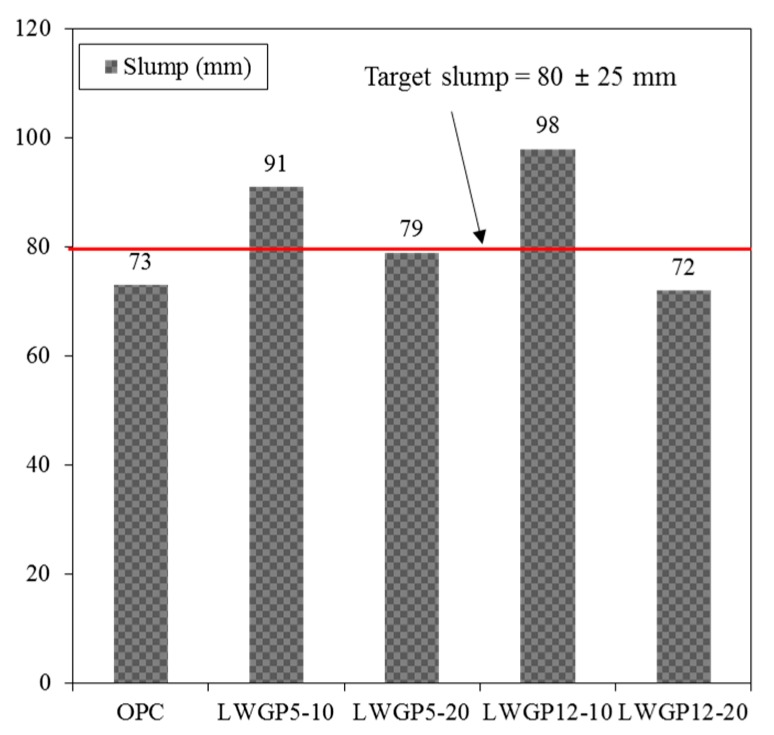
Concrete slump results with the replacement of the LWGP concrete flumes.

Figure 4 shows that slumps less than the target values of 80 ± 25 mm set for construction at a site were obtained. The slump of OPC was 73 mm, thereby meeting the target slump. A high slump was observed at the replacement ratio of 10%, regardless of the cement diameter. In contrast, a low slump was measured at the replacement ratio of 20% because the LWGP had angular and pyramidal shapes; hence, they increased the contact areas with adjacent cement particles, which increased the cohesion and reduced the mobility between them.

### 3.2. Changes in Weight and Volume

**Figure 5:**
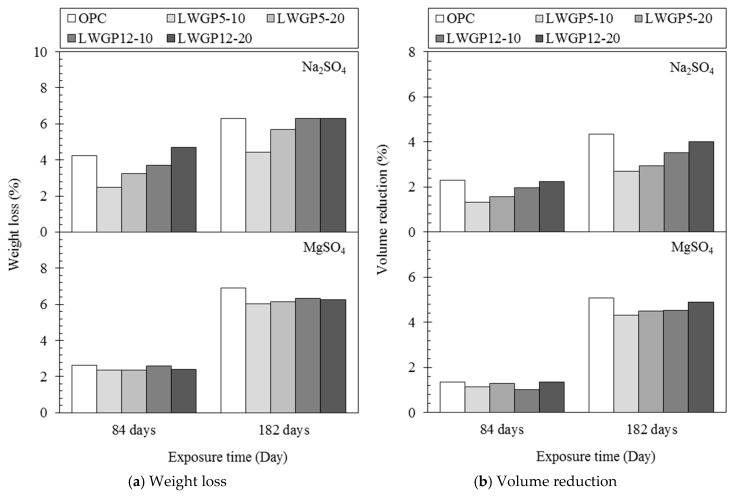
Deterioration of the LWGP-mixed concrete after exposure in different sulfate solutions for 84 and 182 days.

The LWGP-mixed concrete was immersed in a solution of 10% Na_2_SO_4_ and MgSO_4_ for 182 days to evaluate the resistance to sulfate attack by measuring the weight and volume according to ASTM C 1012 [27]. As shown in Figure 5, the blended specimen exhibited a lower reduction ratio of the weight and the volume compared with OPC, regardless of the sulfate type. The influence of the LWGP-mixed concrete was noticeable when Na_2_SO_4_ was used. This phenomenon may be caused by the matrix porosity. The sulfate ions that intruded the concrete initially reacted with calcium hydroxide to form ettringite and gypsum, which continuously accumulated within the pores. In turn, the amounts of hydration products caused the dilatation of the concrete and generating an internal stress around the matrix, followed by cracking. The calcium hydroxide depletion induced the decalcification of the hydration products, thereby resulting in a dissolution of the C–S–H gel and a subsequent increase of the matrix porosity [28,29,30]. Consequently, the flexural and compressive strengths of the concrete structures would gradually be reduced with the increase of the decalcification degradation, along which the matrix structure collapsed. However, according to Yang et al. [31], the rate of the ionic transport is affected by the pore structure, especially the capillaries in the cement matrix. The LWGP-mixed concrete, possessing the lower porosity at the capillaries, may perform the reduced ingress of aggressive ions into the concrete, enhancing the resistance to a sulfate attack resulting from the limited reaction between the sulfates and the hydrates. The higher resistance against sulfate ingress was caused by the reduction of Ca(OH)_2_ because of the pozzolanic reaction obtained from the replacement of the LWGP powder in the concrete. Regardless of the mixture of the LWGP powders, the reduction ratios of the weight and the volume for concretes were smaller when they were immersed in the MgSO_4_ solution than in the Na_2_SO_4_ solution for 182 days. This result can be attributed to the reaction between the Mg^2+^ ions and the hydrates formed in the matrix. According to Lee [4,32], the OH^−^ and Mg^2+^ ions in the pore water reacted to form magnesium hydroxide (Mg(OH)_2_) at early exposure periods, as well as the formation of gypsum and ettringite, filling the voids within the matrix (i.e., micro-filler effect). Therefore, the penetration resistance of ions into the concrete substituted by the LWGP increased with exposure duration to MgSO4 solution, which is validated by the time-dependent behavior of concrete deterioration, implying change of the pore structure. The deterioration by sulfates also decreased at the early ages. However, in the long-term periods, the deterioration of concrete exposed to the MgSO_4_ solution would be accelerated because of an increase of the stress inside the concrete arising from the accumulation of hydrates [33,34]. Furthermore, the C–S–H gel, which is responsible for the concrete strength, can be transformed into the M–S–H gel by the ion exchange caused by the excessive Mg^2+^ ions in the pore solution [34,35]. Consequently, the volume and the weight change ratios for all the specimens immersed in the MgSO_4_ solution showed a higher value at 182 days of exposure compared with that in the Na_2_SO_4_ solution at an identical condition.

### 3.3. Compressive Strength and Strength Change

**Figure 6:**
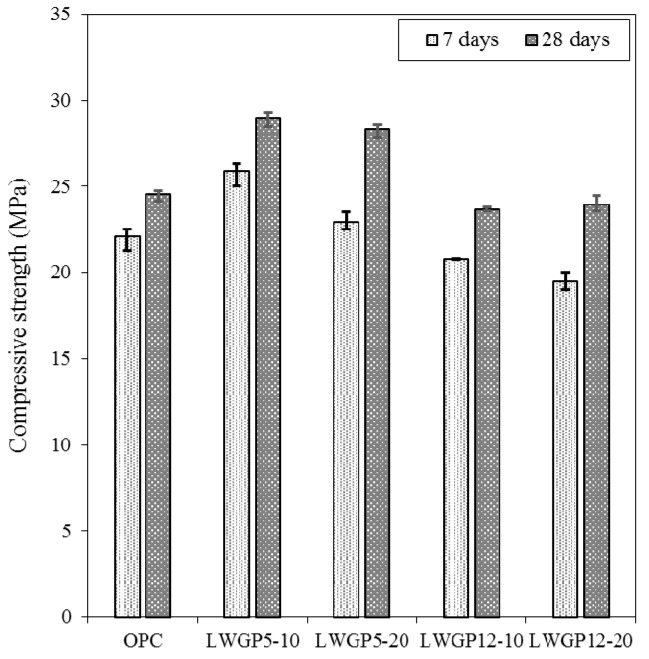
Compressive strength results of the LWGP concrete flumes.

The compressive strengths of the concrete mixed with the LCD powder are shown in Figure 6, in which the compressive strengths were obtained for five cases with different diameters and replacement ratios (i.e., OPC, LWGP5-10, LWGP5-20, LWGP12-10, and LWGP12-20).

Note that the high strengths of concrete at an early age (7 days) were produced when the mean particle diameter of 5 μm with 10% and 20% replacement ratios was used. Similar cases were observed with the mortar test. However, the compressive strengths decreased when the substituting ratio increased to 20%. However, the LWGP12 with large average diameters showed a strength smaller than that of OPC, regardless of the substituting ratios. The LCD cement showed compressive strengths slightly smaller for the diameter of 12 μm; however, the compressive strengths were higher for cement with a 5 μm diameter (Figure 6). This phenomenon may be caused by the fact that the Si and Al ions in the LWGP reacted with the Ca ions, which were included in the pore solution, to form the C–S–H and C–A–H gels, thus producing a dense and more compact matrix [13].

The LWGP5 with small average diameters exhibited a higher strength from 7 days, which resulted in a noticeable strength increase at 28 days, showing the influence of diameters on the strength. When made of finer powders and smaller diameters, the LWGP-mixed concrete could result in an active pozzolanic reaction, leading to an increase in both strength and durability.

**Figure 7:**
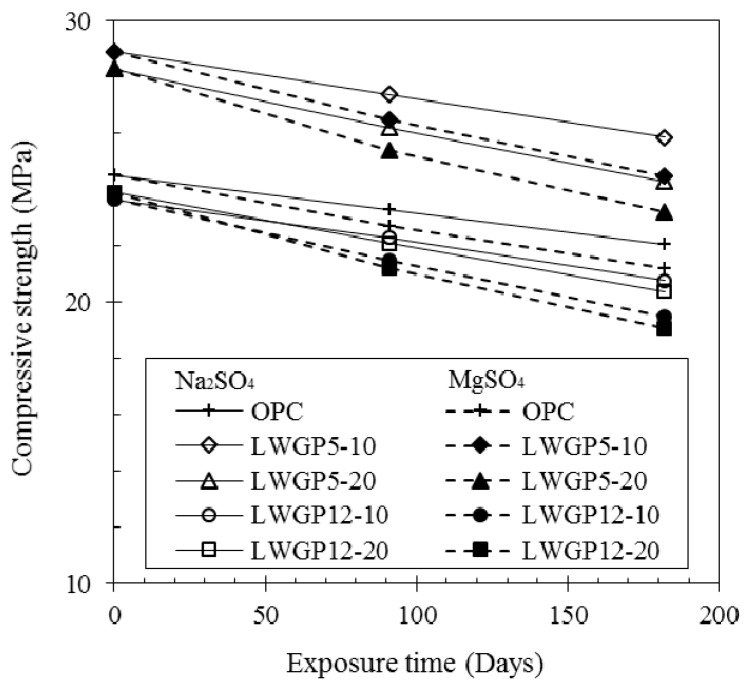
Variation of the compressive strength for the concrete containing LWGP powder under sulfate-bearing environments.

The compressive strengths (KS F 2405 [26]) for the LWGP-mixed concrete exposed to the sulfate solutions were measured to explore the mechanical changes. Figure 7 shows the variation of the compressive strength for the concrete containing LWGP powder under sulfate-bearing environments. The un-bond capping method was used to minimize the measurement errors caused by the surface damage. The reduction ratios of the compressive strength of the LWGP powder, which was smaller compared with that of OPC, were observed under circumstances, including exposure duration and sulfate types. The LWGP powder not only filled the voids physically, but also led to a densification of the cement matrix caused by secondary reactions. This would induce a decreased porosity at the capillary pores and lead to a difference of the resistance from a sulfate attack. A relatively rapid strength decrease was observed because of the additional Mg-based hydration products formed when immersed in the MgSO_4_ solution, leading to a limited strength activation caused by the M–S–H gel formation. The strength reduction trends were similar to those observed with the weight and the volume. The chemical resistance of the concrete exposed to the sea waters and underground waters can be provided by adjusting the replacement ratios.

### 3.4. Pore Distribution of the Concrete Blended with the LWGP Powder

The importance of the pore distribution in the matrix must be expressed. Materials with the same pore size can perform completely differently because of the pore size and dispersion [36,37]. The pore distribution of the blended concretes was measured at 28 days using the MIP, of which the samples (approximately 1.0 cm^3^ in the volume) were obtained from concrete for the compressive strength. The pore size was divided into four groups: 1) Voids: >10 μm; large capillaries: 0.05–10 μm; medium capillaries: 0.01–0.05 μm; and small capillaries: <0.01 μm [38].

**Figure 8:**
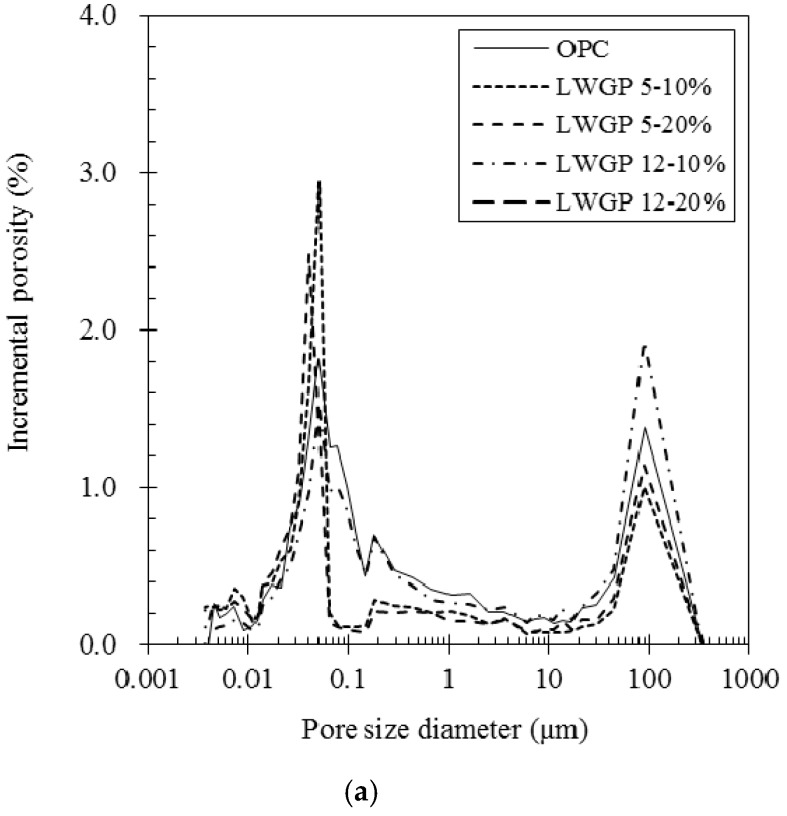

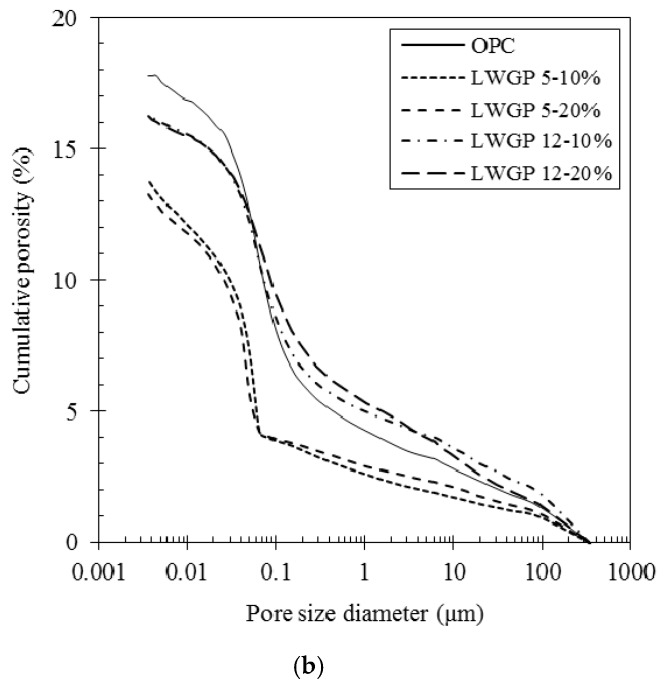
Pore distribution of the specimen obtained from the concrete blended with the LWGP powder. (**a**) The incremental porosity by the pore size diameter; (**b**) The cumulative porosity by the pore size diameter.

The pore decrease of the concrete containing the LWGP powder is elucidated in Table 4 and Figure 8, which show that the pores of OPC of 17.79% are the greatest, followed by the LWGP12-10 (16.32%), LWGP12-20 (16.24%), LWGP5-10 (13.79%), and LWGP5-20 (13.27%). Figure 8a shows the incremental porosity by the pore size diameter of the specimens and Figure 8b shows the cumulative porosity by the pore size diameter of the specimens. The capillary pores were found to be similar to the compressive strength at 28 days because of the pozzolanic reaction by the LWGP. The capillary pores occupied by Ca(OH)_2_ decreased, whereas the gel pores increased by forming the C–S–H gel as the time elapsed. This result led to a pore reduction and a higher strength development [39]. However, both quantitative and qualitative studies on the gel pores, including test and analysis (e.g., nitrogen and helium absorption methods), are necessary for in-depth investigations because the measurement by the MIP adopted herein only provided limited information on the gel pores.

### 3.5. Flexural Capacity

**Figure 9:**
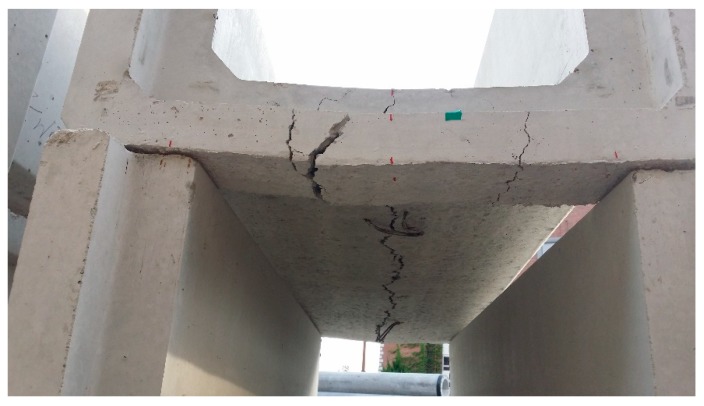
Crack patterns of the LWGP concrete flume.

The structural behavior of the LWGP concrete flumes was investigated by comparing the flexural loads by OPC and LWGP. Testing was done for 28 days based on various diameters and substituting ratios. The cracks were monitored by strain gauges attached to the lower part of the socket. The flexural loads were identified when cracks with a width of 0.05 mm were found at the end of the section (Figure 9). Table 5 and Figure 9 summarize the test results and the crack patterns where the longitudinal cracks were observed, leading to flexural failure of the section. A flexural load greater than 48.5 kN required by KS F 4010 [19] was obtained for all the LWGP concrete flume specimens with a length of 2000 mm.

**Figure 10:**
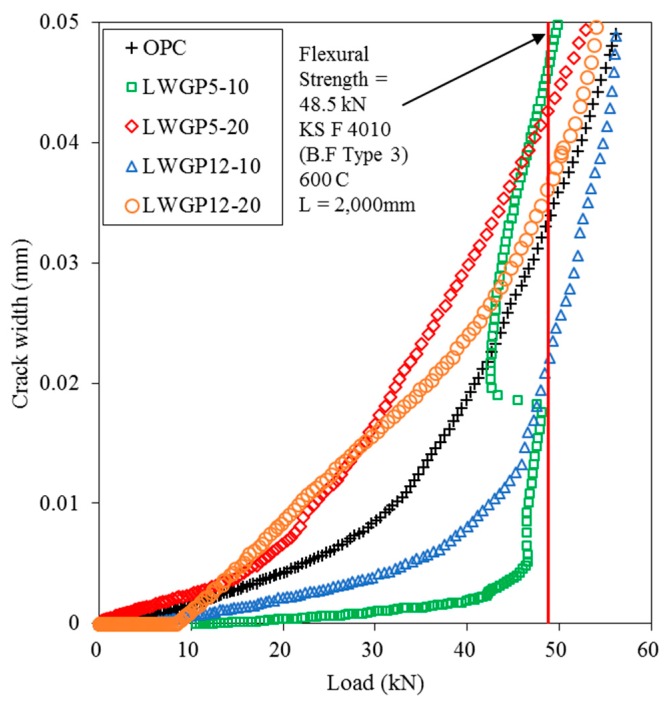
Relationships of the LWGP concrete flume between the crack widths and the loads.

The flexural load of the LWGP concrete flumes greater than that of OPC by 3.5% to 6.5% was observed, demonstrating a trend similar to that of the compressive strength of the LWGP5, which was greater than the compressive strength of OPC by 15% to 17%. Load was applied with 1 mm/min according to KS F 4010 to measure the flexural capacities. The flexural capacity of OPC was 65.66 kN, whereas those of the LWGP concrete flumes were 69.92 kN and 67.99 kN for LWGP5-10 and LWGP5-20, respectively. However, the flexural loads of the LWGP concrete flumes decreased to 62.21 kN and 58.56 kN for LWGP12-10 and LWGP12-20, respectively. Table 5 shows that the flexural capacity decreased when the replacement ratios increased. The pozzolanic reaction contributed to the secondary formation of the C–S–H gel by making the concrete microstructures denser, leading to the increase of the LWGP concrete flume [40]. The LWGP diameters were fine enough to replace OPC; however, the contribution of the flexural load of LWGP12 was smaller than that of LWGP5 because the pozzolanic reaction was not salient. The diameters and substituted ratios should be determined to improve the activity for maximized contribution to the increase of the flexural load and durability of the LWGP. The LWGP with the substituted ratios up to 20% provided an acceptable flexural load and durability. As shown in Figure 10, the initial cracks were effectively controlled for the LWGP5-10 and the LWGP12-10 before the critical loads, at which the cracks rapidly increased. The cracks increased in proportion to the increase of loads for LWGP5-20, LWGP12-20, and OPC.

**Figure 11:**
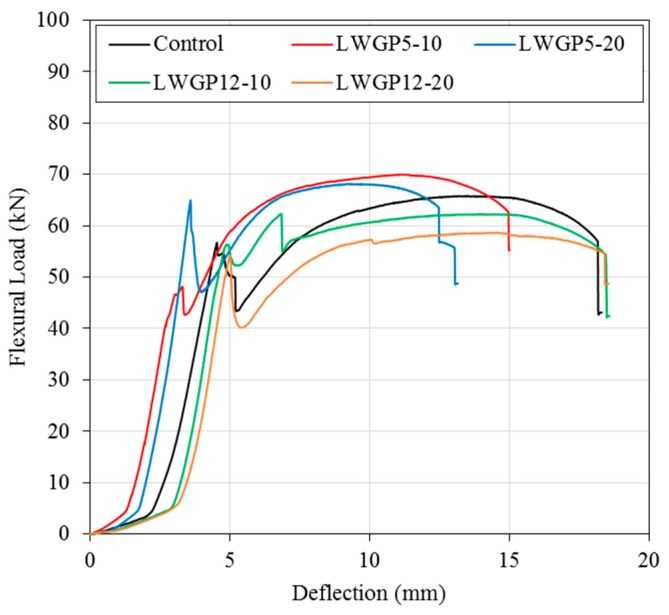
Flexural load–displacement curve of the LWGP reinforced concrete flumes.

**Figure 12:**
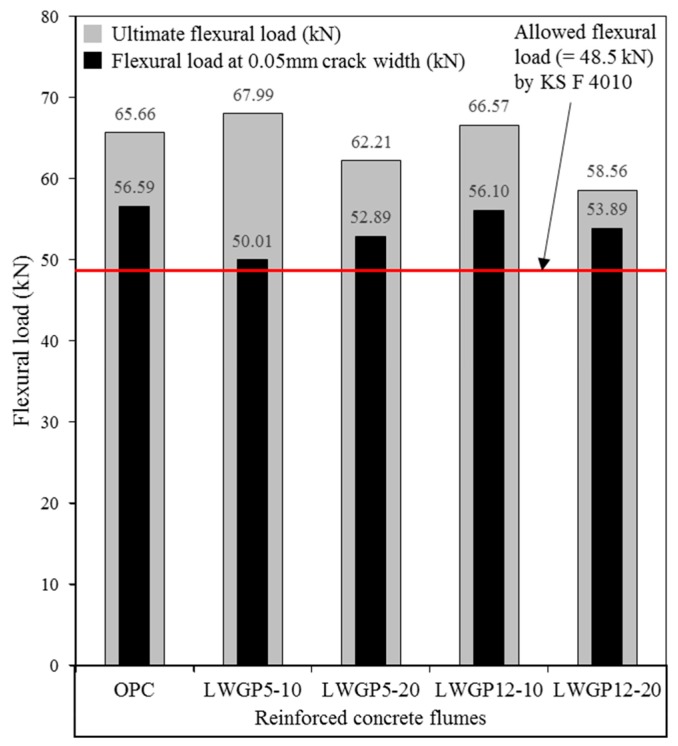
Comparison of the ultimate flexural load of concrete flumes, flexural load of the LWGP concrete flumes at a crack width of 0.05 mm, and the allowable flexural load of standard.

The KS code defines the flexural load of the reinforced concrete flume when the crack width of 0.05 mm was reached to protect the reinforced concrete flume contacted by polluted waters from erosions of wires and structural damages of the concrete. The ductility of the LWGP concrete flume is also shown in Figure 11, where sudden drops of the flexural capacities were observed. From Figure 11, the initial linear stage of the flexural behavior, which is the flexural behavior before cracking (Wcr < 0.05 mm) from the bottom of the LWGP concrete flumes, exhibited a high stiffness caused by the increase in the compressive strength [41]. The deformation of the LWGP concrete flume with the LWGP5 demonstrated a smaller deformation compared to that of the OPC tube, while the yielding point was smaller than that of OPC. A more brittle behavior than that of the OPC tube was also observed (Figure 11) because the wires yielded, causing rapid load drops. However, the flexural loads of the LWGP concrete flumes with a crack width of 0.05 mm were greater than 48.5 kN, which was required by the KS code (Figure 12).

## 4. Conclusions

This study used the LWGP, an industrial waste material, as an admixture to increase the sulfate attack resistance of concrete flumes. The compressive strength, pore structure, weight, volume, and strength change in two kinds of sulfate solution were measured for the durability evaluation. The LWGP was also mixed with the reinforced concrete flumes, and the performance was evaluated by the bending strength test. The results are as follows.

(1) The LWGP yielded large compressive strengths of 23.66 to 28.92 MPa at 28 days. LWGP5 especially resulted in a strength increase greater than those of OPC. By contrast, the strengths of the LWGP12 demonstrated a compressive strength smaller than those of OPC, regardless of the substituted ratios of the LWGP.
(2) A pore decrease of 25.4% compared with OPC was observed at 28 days when the LWGP5-20 powder was used with concrete. The capillary pores (below 10 μm) related with the ion penetrations were found in the order of OPC (17.79%), LWGP12-10 (16.32%), LWGP12-20 (16.24%), LWGP5-10 (13.79%), and LWGP5-20 (13.27%). The capillary pores occupied by Ca(OH)_2_ decreased, whereas the gel pores increased by forming the C–S–H gel. This led to pore reduction and a higher strength development.
(3) The reduction ratios of the weight and the volume for concretes with the mixture of LWGP powders were smaller than with OPC. The reduction ratios of the weight and the volume of the LWGP5-10 concrete were the lowest as 4.41% and 2.70% when they were immersed in the Na_2_SO_4_ solution for 182 days, whereas those were 6.04% and 4.32% when immersed in the MgSO_4_ solution. This trend was found to be similar to that of the compressive concrete strength immersed in the sulfate solution.
(4) The maximum flexural load of the LWGP concrete flume was the highest at 69.92 kN in the LWGP5-10 contained concrete, while the LWGP10 contained concrete flumes smaller than the OPC concrete flumes. The flexural load of the concrete was generally increased or decreased in proportion to the compressive strength; hence, the tendency similarity was confirmed. Moreover, the opposite case was that the decrease of the flexural load of the concrete flume with LWGP10 also had the same tendency.
(5) The flexural loads of all the LWGP concrete flumes with a crack width of 0.05 mm were greater than the 48.5 kN required by the KS code, but smaller than the flexural loads of OPC. The initial cracks were effectively controlled for LWGP5-10 and LWGP12-10 before the critical load.
(6) The durability of the conventional reinforced concrete flumes against the sulfate attack resistance was improved by substituting cement by LWGP, thereby extending the use of the new reinforced concrete flumes to the replacement of conventional reinforced concrete flumes. The applicability was also validated by the flexural test complying with KS.

## Figures and Tables

**Table 1:** Chemical and physical properties of binders.

	Oxide Composition								Specific Gravity
	CaO	SiO_2_	Al_2_O_3_	Fe_2_O_3_	SO_3_	Na_2_O	K_2_O	MgO	
OPC	65.42	19.09	4.06	3.11	3.79	0.10	1.58	2.85	3.15
LWGP5	10.00	66.90	18.21	0.08	–	0.23	0.05	0.44	2.79
LWGP12	10.3	65.7	18.0	0.14	–	0.18	0.04	0.42	2.81

**Table 2:** Physical properties of the fine and coarse aggregates.

	Fineness Modulus	Water Absorption* (%)	Specific Gravity (N/m^3^)
Fine aggregate	2.94	1.3	2.56
Coarse aggregate	6.51	1.2	2.59

* Fine and coarse aggregates were immersed in water for 24 ± 4 hours to fully saturate the pores, according to ASTM C127-15 and ASTM C128-15 [23,24].

**Table 3:** Mixing proportions of the reinforced concrete flume with the LWGP.

W/B (%)	S/a (%)	Unit Weight (kg/m^3^)					
		Water	Cement	LWGP5, LWGP12		Fine Aggregate	Coarse Aggregate
47.2	39.6	150	360	0%	0	400	1109
			324	10%	36		
			288	20%	72		

**Table 4:** Comparison of porosity by the pore size of LWGP concrete specimen at 28 days.

Porosity		28 days				
		OPC	LWGP 5-10	LWGP 5-20	LWGP 12-10	LWGP 12-20
Void	> 10 μm	2.88	1.76	2.17	3.69	3.41
Large-capillary	0.05–10 μm	9.74	5.41	3.41	8.49	9.11
Medium-capillary	0.01–0.05 μm	4.30	5.07	6.33	3.46	3.01
Small-capillary	< 0.01μm	0.87	1.56	1.36	0.69	0.72
Total		17.79	13.79	13.27	16.32	16.24

**Table 5:** Compressive and flexural load results with the crack width.

	Flexural Loads of the Reinforced Concrete Flumes			
	P_u_(kN)	P_cr_(kN)	W_cr_(mm)	Normalized P_cr_/W_cr_(%)
OPC	65.66	56.59	0.0501	100
LWGP5-10	69.92	50.01	0.0504	87.85
LWGP5-20	67.99	52.89	0.0507	92.36
LWGP12-10	62.21	56.10	0.0505	98.35
LWGP12-20	58.56	53.89	0.0508	93.92
